# Regulation of AmpC-Driven β-Lactam Resistance in Pseudomonas aeruginosa: Different Pathways, Different Signaling

**DOI:** 10.1128/mSystems.00524-19

**Published:** 2019-12-03

**Authors:** Gabriel Torrens, Sara Belén Hernández, Juan Alfonso Ayala, Bartolome Moya, Carlos Juan, Felipe Cava, Antonio Oliver

**Affiliations:** a Servicio de Microbiología and Unidad de Investigación, Hospital Son Espases, Instituto de Investigación Sanitaria de Baleares (IdISBa), Palma, Spain; b Laboratory for Molecular Infection Medicine Sweden, Department of Molecular Biology, Umeå Centre for Microbial Research, Umeå University, Umeå, Sweden; c Departamento de Virología y Microbiología, Centro de Biología Molecular Severo Ochoa, Madrid, Spain; d Department of Pharmaceutics, College of Pharmacy, University of Florida, Orlando, Florida, USA; University of California, Irvine

**Keywords:** AmpC β-lactamase, *Pseudomonas aeruginosa*, muropeptide, peptidoglycan

## Abstract

The hyperproduction of the chromosomal AmpC β-lactamase is the main mechanism driving β-lactam resistance in Pseudomonas aeruginosa, one of the leading opportunistic pathogens causing nosocomial acute and chronic infections in patients with underlying respiratory diseases. In the current scenario of the shortage of effective antipseudomonal drugs, understanding the molecular mechanisms mediating AmpC hyperproduction in order to develop new therapeutics against this fearsome pathogen is of great importance. It has been accepted for decades that certain cell wall-derived soluble fragments (muropeptides) modulate AmpC production by complexing with the transcriptional regulator AmpR and acquiring different conformations that activate/repress *ampC* expression. However, these peptidoglycan-derived signals have never been characterized in the highly prevalent P. aeruginosa stable AmpC hyperproducer mutants. Here, we demonstrate that the previously described fragments enabling the transient *ampC* hyperexpression during cefoxitin induction (1,6-anhydro-*N*-acetylmuramyl-pentapeptides) also underlie the *dacB* (penicillin binding protein 4 [PBP4]) mutation-driven stable hyperproduction but differ from the 1,6-anhydro-*N*-acetylmuramyl-tripeptides notably overaccumulated in the *ampD* knockout mutant. In addition, a simultaneous greater accumulation of both activators appears linked to higher levels of AmpC hyperproduction, although our results suggest a much stronger AmpC-activating potency for the 1,6-anhydro-*N*-acetylmuramyl-pentapeptide. Collectively, our results propose a model of AmpC control where the activator fragments, with qualitative and quantitative particularities depending on the pathways and levels of β-lactamase production, dominate over the repressor (UDP-*N*-acetylmuramyl-pentapeptide). This study represents a major step in understanding the foundations of AmpC-dependent β-lactam resistance in P. aeruginosa, potentially useful to open new therapeutic conceptions intended to interfere with the abovementioned cell wall-derived signaling.

**IMPORTANCE** The extensive use of β-lactam antibiotics and the bacterial adaptive capacity have led to the apparently unstoppable increase of antimicrobial resistance, one of the current major global health challenges. In the leading nosocomial pathogen Pseudomonas aeruginosa, the mutation-driven AmpC β-lactamase hyperproduction stands out as the main resistance mechanism, but the molecular cues enabling this system have remained elusive until now. Here, we provide for the first time direct and quantitative information about the soluble cell wall-derived fragments accounting for the different levels and pathways of AmpC hyperproduction. Based on these results, we propose a hierarchical model of signals which ultimately govern *ampC* hyperexpression and resistance.

## Abstract (precis)

The extensive use of β-lactam antibiotics and the bacterial adaptive capacity have led to the apparently unstoppable increase of antimicrobial resistance, one of the current major global health challenges. In the leading nosocomial pathogen Pseudomonas aeruginosa, the mutation-driven AmpC β-lactamase hyperproduction stands out as the main resistance mechanism, but the molecular cues enabling this system have remained elusive until now. Here, we provide for the first time direct and quantitative information about the soluble cell wall-derived fragments accounting for the different levels and pathways of AmpC hyperproduction. Based on these results, we propose a hierarchical model of signals which ultimately govern *ampC* hyperexpression and resistance.

## INTRODUCTION

Pseudomonas aeruginosa stands out among the human opportunistic pathogens, since it is the primary cause of ventilator-associated pneumonia and burn wound infections (1, 2) along with chronic respiratory infections in patients with chronic underlying diseases (3–5). Among its well-known plethora of resources for antibiotic resistance, the hyperproduction of the intrinsic inducible cephalosporinase AmpC is the main mechanism used by this pathogen to cope with β-lactams (6–8).

The regulation of AmpC β-lactamases was initially studied in other Gram-negative bacteria harboring this type of intrinsic enzyme, such as Citrobacter freundii and Enterobacter cloacae. It was proposed that the expression of these β-lactamases was controlled by the LysR-type AmpR transcriptional regulator complexed with certain cell wall (peptidoglycan)-derived fragments, generically known as muropeptides (9–12). As in other species, P. aeruginosa *ampR* and *ampC* genes form a divergent operon with overlapping promoters and with the fragment in between acting as an AmpR binding site (13). It was proposed that upon muropeptide binding, AmpR would change its conformation to modulate RNA polymerase activity and thus *ampC* transcription (11, 14–16). Further, this mechanism was shown to be intimately linked to the peptidoglycan recycling biology. During growth, the bacterial cell wall needs to expand, and peptidoglycan is cleaved on each generation by hydrolytic enzymes to allow new material insertion and cell division. Although part of the cleaved peptidoglycan fragments are released to the extracellular medium, most of them are transported into the cytoplasm through the AmpG permease for recycling (17–21). Once in the cytosol, the muropeptides (mainly *N*-acetylglucosamine-1,6-anhydro-*N*-acetylmuramyl-peptides [NAG-anhNAM-peptides]) are further processed by NagZ, which removes the *N*-acetylglucosamine residue (NAG) to produce 1,6-anhydro-*N*-acetylmuramyl-peptides (anhNAM-peptides), and by the amidase AmpD, which cleaves the bond between the anhNAM and the stem peptides (22, 23). The concept of recycling precisely comes from the fact that the resulting monosaccharides (NAG and anhNAM) and peptides are reused to assemble an essential unit for peptidoglycan synthesis: the uridine 5′-pyrophosphoryl-*N*-acetylmuramic acid-pentapeptide (UDP-NAM-P5) (13, 19–21, 24, 25). Under normal conditions, the UDP-NAM-P5 units bind to AmpR, and this complex is believed to repress *ampC* expression to basal levels. This is the reason why UDP-NAM-P5 has been known as an AmpC repressor (13, 16, 17). Conversely, when bacteria are exposed to the so-called inducer β-lactams (e.g., cefoxitin), the derived inhibition of certain penicillin binding proteins (PBPs), such as PBP4 (encoded by *dacB*), alters the peptidoglycan turnover balance in favor of autolysis (16, 17, 26–31). It is believed that this situation boosts the amount of muropeptides reaching the cytosol, which saturate NagZ and AmpD activities, providing an increased quantity of NAG-anhNAM-peptides and/or anhNAM-peptides that displace the repressor UDP-NAM-P5 from AmpR (32, 33). Then, the new AmpR-muropeptide complex promotes *ampC* hyperexpression entailing resistance to hydrolyzable β-lactams such as cefoxitin (7, 16, 17, 25, 31, 34). Besides this transient induction, the selection of mutations leading to stable hyperproduction is a very common event among clinical strains, obviously due to the pressure exerted by β-lactam treatments. Among the different mutations that enable AmpR-dependent AmpC hyperproduction (7, 13, 24, 25, 34–36), those on *ampD* and *dacB* are the most frequently reported in P. aeruginosa (12, 37–41).

As stated before, while certain peptidoglycan-derived fragments (known as activators) were described to enable AmpR-mediated AmpC hyperproduction decades ago in different species (13, 16, 17, 42, 43), the identification of NAG-anhNAM-P5 and its derivative anhNAM-P5 as the drivers for the transient AmpC induction in P. aeruginosa is recent (33). Other works, based on *in vitro* experiments, have suggested that only muropeptides containing a terminal d-Ala-d-Ala motif (i.e., muropentapeptides) bind AmpR for *ampC* induction (32, 44). Thus, these results contrast with other work in different Gram-negative bacteria, such as C. freundii (16, 17), E. cloacae (43), Aeromonas hydrophila (45), or Stenotrophomonas maltophilia (46), where different AmpC-activating signals (murotripeptides [anhNAM-P3] and murotetrapeptides [anhNAM-P4]) were reported. However, the signaling for AmpC hyperproduction in P. aeruginosa strains bearing the mentioned mutations in *dacB* or *ampD* (8, 37, 47) and thus of extraordinary clinical relevance has never been ascertained. Some clues could be deduced from the work with AmpD mutants in other species (16, 17, 42, 43), but today there are no data regarding the signals sustaining the highly prevalent *dacB* mutational pathway.

Given these important gaps in the knowledge regarding P. aeruginosa AmpC regulation, and the current scenario of increasing prevalence of its resistance in health care settings (48–51), deciphering the signals enabling stable AmpC hyperproduction in this species becomes crucial. In this work, we have performed a comprehensive and quantitative analysis of soluble muropeptides in diverse mutant backgrounds to identify the signals governing AmpC hyperproduction. Our results revealed a very particular dynamic of AmpC regulation for P. aeruginosa, in which the AmpC production level depends on the nature and quantity of cell wall-derived activators. Thus, this work is a major step toward understanding the basis of P. aeruginosa AmpC-mediated hyperproduction, an essential instrument to conceive future therapies intended to interfere with the involved signaling for combating β-lactam resistance.

## RESULTS

### AmpC hyperproduction in Pseudomonas aeruginosa: different pathways, different signaling.

**FIG 1:**
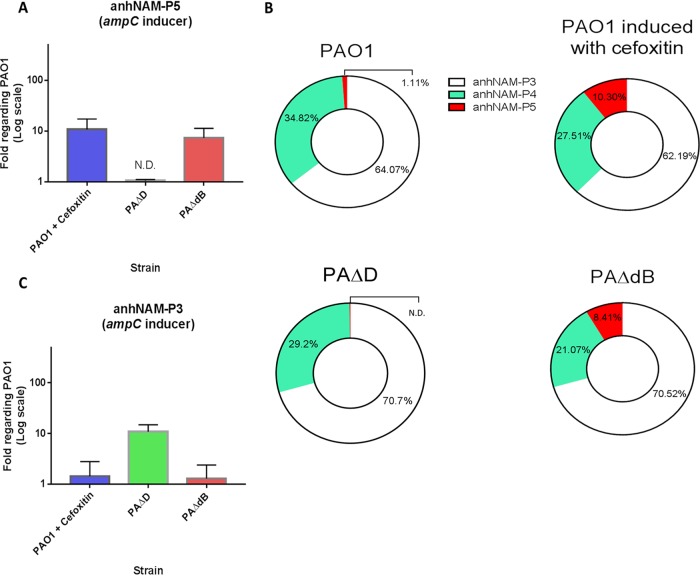
Accumulation of the AmpC activator anhNAM-muropeptides in the cefoxitin-induced PAO1 and in hyperproducer mutants of Pseudomonas aeruginosa. (A and C) The columns represent the mean values of anhNAM-P5 (A) and anhNAM-P3 (C), compared to PAO1, from three independent determinations, and the error bars show the standard deviation (SD). (B) The different-colored sections within the circles represent the proportion of each muropeptide with regard to the total amount of soluble anhNAM-peptides of each strain; the data were extracted from the cited three independent experiments. ND, not detected.

The ultrahigh-pressure liquid chromatography–mass spectrometry (UPLC-MS) analyses of soluble intracellular muropeptides in the wild-type strain PAO1 (Fig. 1) showed that the most abundant molecules within the total anhNAM-peptide pool were anhNAM-P3 (ca. 65%) followed by anhNAM-P4 (ca. 35%) and, at a great distance, by anhNAM-P5 (ca. 1%). However, the amount of this last muropeptide increased ca. 10-fold upon cefoxitin treatment (Fig. 1A and B). Interestingly, the levels of accumulated anhNAM-P5 in the *dacB* mutant (PAΔdB) were similar to those of cefoxitin-induced PAO1 (7.3- ± 2.9-fold compared to PAO1 [Fig. 1A and B]). As can be observed in Fig. 1B, this anhNAM-P5 increase appeared in exchange of a notable reduction in the proportion of anhNAM-P4 within the respective anhNAM-peptide pool (the proportion of anhNAM-P3 remaining stable), which suggests that the total anhNAM-peptide amount both in cefoxitin-induced PAO1 and in PAΔdB is similar to that of PAO1 and that anhNAM-P4 does not play an important role to promote *ampC* expression in these strains.

Besides anhNAM-P5, there was a significant increase in the accumulation of its parent molecule, NAG-anhNAM-P5, in the cefoxitin-induced PAO1 and PAΔdB, compared to PAO1 (ca. 5-fold [data not shown]). However, since NagZ allows the cytosolic cleavage of NAG from NAG-anhNAM-peptides, it is understandable that the accumulation of anhNAM-peptides is proportional to the amount of NAG-anhNAM-peptides reaching the cytosol. For these reasons, in this study we have focused on anhNAM-peptide quantification, a conception supported by previous works (42, 43), in which anhNAM-P5 was considered the genuine AmpR-binding signal.

In contrast to PAΔdB, the *ampD* mutant (PAΔD) showed levels of anhNAM-P5 (Fig. 1A and B) below the wild type and even below the detection limit. Conversely, the intracellular accumulation of anhNAM-P3 increased ca. 10-fold in PAΔD compared to PAO1 (10.9 ± 3.0 [Fig. 1C]). Since anhNAM-P3 is the most abundant chemical species within the anhNAM-peptide pool under normal conditions (ca. 65% [Fig. 1B]), an increase of 10-fold in this muropeptide accumulation necessarily entails a significant increase in the total amount of soluble anhNAM-peptides in PAΔD. Moreover, given that the anhNAM-P5 accumulation (Fig. 1A and B) was under the detection limit in PAΔD, it seems plausible that the amount of this chemical species remained at wild-type levels, thus losing mass within the total pool. These facts suggest that anhNAM-P5 does not significantly sustain AmpC hyperproduction in PAΔD.

### Simultaneous accumulation of 1,6-anhydro-*N*-acetylmuramyl-tripeptides and -pentapeptides drives high-level AmpC hyperproduction.

**FIG 2:**
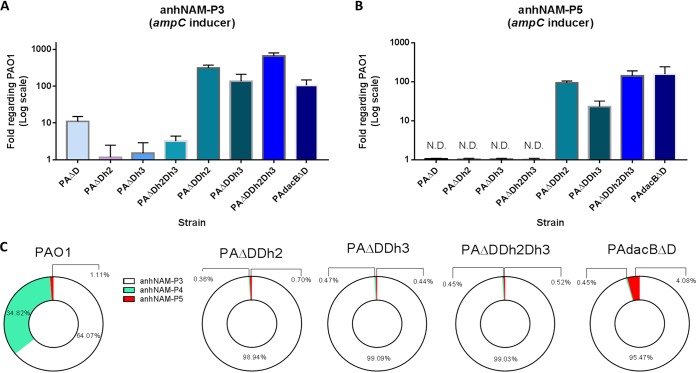
Accumulation of the AmpC activator anhNAM-muropeptides in high-level hyperproducer mutants of Pseudomonas aeruginosa. (A and B) The columns represent the mean values of anhNAM-P3 (A) and anhNAM-P5 (B) (both compared to PAO1 values), from three independent determinations, and the error bars show the SD. (C) The different-colored sections within the circles represent the proportion of each muropeptide with regard to the total amount of soluble anhNAM-peptides of each strain; the data were extracted from the cited three independent experiments. ND, not detected.

To better understand the role of anhNAM-P3 and anhNAM-P5 as AmpC activators, we intracellularly quantified them in a panel of mutants displaying increasing levels of AmpC production. This panel included single knockout mutants in the periplasmic amidases AmpDh2 (PAΔDh2) and AmpDh3 (PAΔDh3) (52–55) and different combinations among them and/or with the abovementioned *ampD* or *dacB* deletions (Table 1; Fig. 2). As expected, we observed no significant differences in the level of anhNAM-P3 or anhNAM-P5 accumulation between PAΔDh2 or PAΔDh3 and PAO1 (Fig. 2A and B), since these mutants show basal *ampC* expression (Table 1) (56). However, the anhNAM-P3 (but not anhNAM-P5) level increased ca. 3 times in the double mutant PAΔDh2Dh3, consistent with a slight but significant increase of its *ampC* expression (ca. 2-fold compared to PAO1 [Table 1]). Interestingly, a simultaneous overaccumulation of both anhNAM-P3 and anhNAM-P5 was observed in the PAΔDDh2, PAΔDDh3, PAΔDDh2Dh3, and PAdacBΔD strains (Fig. 2A and B), all of them displaying high levels of *ampC* expression (Table 1) (47, 56, 57). Particularly, the two highest AmpC-hyperproducing strains, PAΔDDh2Dh3 and PAdacBΔD (both with *ampC* mRNA levels over 1,000-fold higher than PAO1), showed an increase of ca. 600-fold (655- ± 141-fold) of anhNAM-P3 and almost 150-fold of anhNAM-P5 and of around 100-fold (105- ± 42-fold) of anhNAM-P3 and 160-fold (160- ± 40-fold) of anhNAM-P5, respectively, compared to PAO1 (Fig. 2A and B). Finally, and similarly to what has been mentioned above for PAΔD, our results suggest that an important increase in the total anhNAM-peptide pool must exist in the high-level AmpC-hyperproducing mutants, since an increase of more than 100-fold with regard to wild type in the most abundant anhNAM-peptide (anhNAM-P3) should necessarily affect the whole (Fig. 2C). Additionally, the fact that the proportion of anhNAM-P5 was barely reduced (or even increased in PAdacBΔD) in this set of mutants within the anhNAM-peptide pool (Fig. 2C) supports the net increase of this species compared to wild-type level (Fig. 2B), whereas the drastic reduction of anhNAM-P4 within the whole indicates that this species remained at wild-type levels, if not below.

**TABLE 1:** Strains used in this work, displayed with their features related to the profile of β-lactam resistance (ceftazidime MIC and *ampC* expression)

Strain	Genotype/relevant characteristic(s)	Ceftazidime MIC (mg/liter)	*ampC* mRNA expression, mean ± SD^*a*^	Source or reference
PAO1^*b*^	Completely sequenced reference strain	1	1	Laboratory collection
PAΔD	PAO1 Δ*ampD*::*lox*; AmpD is an *N*-acetyl-anhydromuramyl-l-alanine amidase involved in peptidoglycan recycling; negative regulator of AmpC expression	8	47 ± 9.5	56
PAΔDh2	PAO1 Δ*ampDh2*::*lox*; AmpDh2 is an additional AmpD homologue of P. aeruginosa	0.75	1.1 ± 0.2	56
PAΔDh3	PAO1 Δ*ampDh3*::*lox*; AmpDh3 is an additional AmpD homologue of P. aeruginosa	1	1.2 ± 0.3	56
PAΔDh2Dh3	PAO1 Δ*ampDh2*::*lox* Δ*ampDh3*::*lox*	0.75	2.3 ± 0.14	56
PAΔDDh2	PAO1 Δ*ampD*::*lox* Δ*ampDh2*::*lox*	12	79.5 ± 12.2	56
PAΔDDh3	PAO1 Δ*ampD*::*lox* Δ*ampDh3*::*lox*	48	251.2 ± 51.9	56
PAΔDDh2Dh3	PAO1 Δ*ampD*::*lox* Δ*ampDh2*::*lox* Δ*ampDh3*::*lox*; mutant derepressed for AmpC production	48	1,225 ± 101	56
PAΔdB	PAO1 Δ*dacB*::*lox; dacB* encodes the nonessential penicillin-binding protein 4	24	51 ± 16	57
PAdacBΔD	1A1 spontaneous *dacB* knockout mutant (W273X) of PAO1; *ampD*::*lox*; mutant derepressed for AmpC production	96	1,770 ± 401	57
PAΔAG	PAO1 Δ*ampG*::*lox*; *ampG* encodes the specific permease allowing the entry of certain muropeptides into the cytosol	1	1.1 ± 0.4	47
PAΔDDh2Dh3ΔAG	PAO1 Δ*ampD*::*lox* Δ*ampDh2*::*lox* Δ*ampDh3*::*lox* Δ*ampG*::*lox*	1	0.95 ± 0.2	74
PAdacBΔDG	1A1 spontaneous *dacB* knockout mutant (W273X) of PAO1; *ampD*::*lox ampG*::*lox*	0.75	1.7 ± 0.6	47

a Data obtained from the indicated references.

b The *ampC* mRNA level in PAO1 under cefoxitin induction was 50 ± 14 with regard to basal conditions.

### AmpG disruption reduces the intracellular accumulation of 1,6-anhydro-*N*-acetylmuramyl-peptides, preventing AmpC hyperproduction.

**FIG 3:**
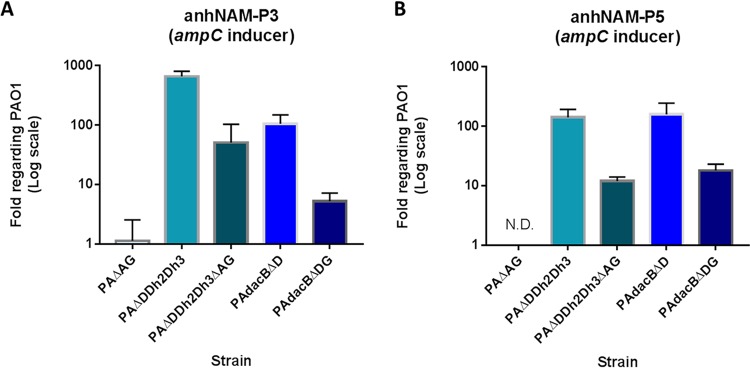
Accumulation of the AmpC activator anhNAM-muropeptides in mutants of Pseudomonas aeruginosa with impaired peptidoglycan recycling through AmpG disruption. The columns represent the mean values of anhNAM-P3 (A) and anhNAM-P5 (B), compared to PAO1, from three independent determinations, and the error bars show the SD. ND, not detected.

The results displayed in Fig. 3A and B confirm that *ampG* deletion drastically decreases the intracellular accumulation of anhNAM-P3 and anhNAM-P5 in P. aeruginosa. This can be observed in the comparison of PAO1 with PAΔAG, in which the amount of these signals was barely quantifiable, but also in PAΔDDh2Dh3ΔAG and PAdacBΔDG. In these two mutants, which proceed from PAΔDDh2Dh3 and PAdacBΔD (high-level AmpC hyperproducers), respectively, the AmpG disruption not only abolished AmpC hyperproduction (Table 1) but also significantly reduced the amount of both activator signals, between 10- and 15-fold with regard to their parent strains.

### The levels of UDP-*N*-acetylmuramic acid-pentapeptide are not fully proportional to AmpC production but respond to peptidoglycan recycling blockade.

**FIG 4:**
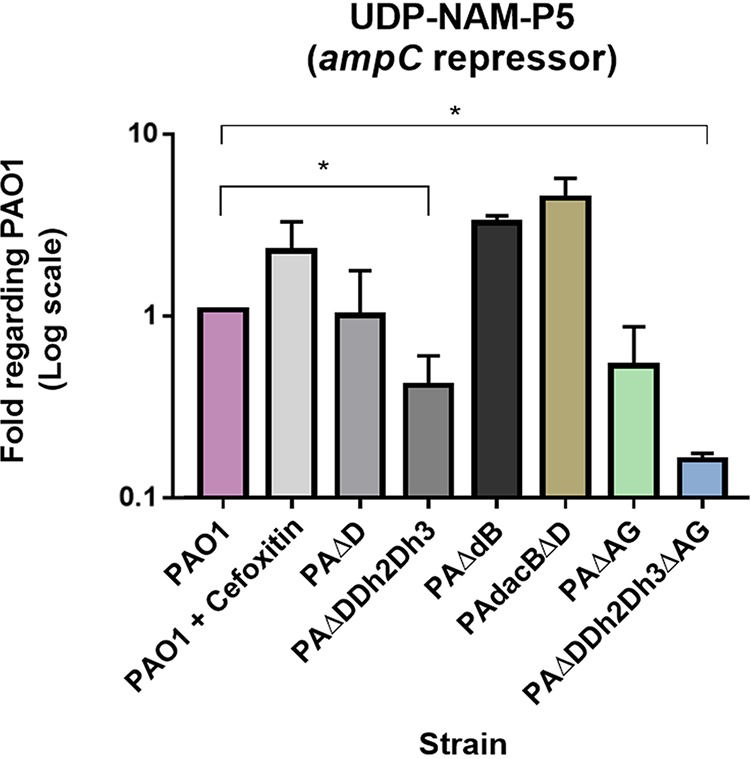
Quantification of the AmpC repressor (UDP-NAM-P5), in different hyperproducer and/or peptidoglycan-recycling-impaired strains of Pseudomonas aeruginosa. The columns represent the mean values of UDP-NAM-P5 with regard to PAO1 (considered 1), obtained from three independent determinations, and the error bars show the SD. *, Student’s *t* test *P* value of <0.05.

The results shown in Fig. 4 indicate that the variations in the amount of the alleged AmpC repressor UDP-NAM-P5 are much smaller (approximately ranging from 0.5- to 5-fold compared to wild type) than those of the AmpC-activating signals among our strains. Furthermore, the accumulation of UDP-NAM-P5 was not consistently inversely proportional to the *ampC* expression levels (Table 1). In fact, a clear inverse correlation between AmpC production and UDP-NAM-P5 levels was seen only for PAΔDDh2Dh3, and thus, the amount of this repressor was variable in the rest of the strains and even greater than that of PAO1 in some cases, such as PAΔdB, PAdacBΔD, or the cefoxitin-induced PAO1. In contrast, its amount was consistently reduced (even below half the wild-type value) in the strains in which the peptidoglycan recycling was seriously impaired, such as those containing the triple AmpD homologues and/or AmpG disruptions (Fig. 4).

## DISCUSSION

Through the present study, we have identified for the first time the overaccumulation of anhNAM-P5 as the activator signal underlying the PBP4 mutational route of AmpC hyperproduction in P. aeruginosa, the most frequent mechanism causing AmpC-mediated resistance in clinical strains (37). Therefore, this pathway shares the same signal that was previously proposed to mediate the cefoxitin induction (33), which is also supported by our results (Fig. 1). In contrast, our observations indicate that anhNAM-P3 is the main signal accounting for AmpC hyperproduction in the AmpD inactivation-mediated pathway, also very frequently found in P. aeruginosa of clinical origin. This would be in agreement with previous studies that showed increased anhNAM-P3 accumulation in *ampD*-defective mutants of other Gram-negative species (16, 17, 42, 43). Thus, our results suggest that the mild levels of AmpC hyperproduction (*ampC* mRNA ca. 50-fold higher than PAO1 [56, 57]), like those shown by the cefoxitin-induced PAO1, PAΔD, or PAΔdB (Table 1), can be achieved through increased levels of one of the two different activators, anhNAM-P3 or anhNAM-P5. However, our results also indicate that anhNAM-P5 has a much stronger AmpC-activating capacity, given that with a substantially smaller amount than that of anhNAM-P3, it triggers a similar *ampC* hyperexpression. In other words, anhNAM-P3 could play the same activator role but requiring a much larger number of molecules. Therefore, although our results do not support the full need for a terminal d-Ala-d-Ala in the stem peptide of the cell wall-derived signal for *ampC* expression promotion, they suggest a much higher capacity for AmpR binding if this moiety is present, in agreement with previous works (32).

The mentioned increased accumulation of anhNAM-P3 in PAΔD can be explained by the facts that, under regular conditions, (i) most of the released muropeptides taken up through AmpG have tetrapeptide stems, given that the d,d-transpeptidases (e.g., high-molecular-mass PBPs) and transpeptidation-independent d,d-carboxypeptidases readily cleave the terminal d-Ala from the pentapeptide stems in the sacculus (18, 33, 58, 59); (ii) NAG from NAG-anhNAM-P4 is efficiently cleaved by NagZ (42, 43); and (iii) the terminal d-Ala from anhNAM-P4 is largely trimmed by the cytosolic l,d-carboxypeptidase LdcA (60–62). Finally, the AmpD disruption would disable the cytosolic cleavage to release the ahnNAM from the stem tripeptide, thereby increasing the amount of the activator anhNAM-P3 (Fig. 1C). Accordingly, given that AmpD should not affect the periplasmic turnover and stem peptide length but rather the cytosolic processing of muropeptides, the very low anhNAM-P5 level in PAΔD seems also logical (Fig. 1A and B).

Conversely, cefoxitin causes an increase in the proportion of pentapeptides within the murein sacculus through the inhibition of the d,d-carboxypeptidase activities of the low-molecular-mass PBPs such as PBP4 (as shown in *A. hydrophila* and P. aeruginosa, for instance [31, 45, 63–66]). This inhibitory effect is believed to move the turnover balance toward an increased autolysis (16, 17, 26, 28, 29, 31), leading to a rise in the released NAG-anhNAM-P5 and anh-NAM-P5 (16, 17, 28, 31, 67), which would ultimately saturate AmpD and bind AmpR to induce *ampC* expression (33) (Fig. 1A and B). In this regard, our results suggest that *dacB* inactivation mimics the cefoxitin induction in P. aeruginosa; in fact, an increase in the level of stem pentapeptides in the peptidoglycan is observed when *dacB* is deleted together with other low-mass PBPs (59). Further, the inactivation of PBP4 would obviously resemble the abovementioned cefoxitin effects, causing a higher peptidoglycan autolysis coresponsible for the increased amount of cytosolic anhNAM-P5. These ideas are supported by the fact that both cefoxitin induction and PBP4 deletion have been shown to cause the activation of the CreBC two-component system in contrast with AmpD disruption, which has no effect on this system (53, 68, 69). CreBC activation has pleiotropic effects, among them the documented increase in the β-lactam resistance output derived from a given AmpC production level, which evidences its relevance (13, 53, 69–71). Thus, our results suggest that the abnormal overaccumulation of soluble anhNAM-P5 would be the driver of the role of PBP4 as cell wall damage sentinel and the signal detected by the membrane-bound CreC sensor leading to the activation of CreBC on one hand and to the promotion of *ampC* expression (via AmpR binding) on the other (57, 68).

With regard to the high-level AmpC hyperproduction, our results clearly demonstrate a concomitant overaccumulation of anhNAM-P3 and anhNAM-P5 in the strains with this profile (ca. 100- to 1,000-fold of *ampC* mRNA compared to PAO1), regardless of the pathway: PAΔDDh2, PAΔDDh3, PAΔDDh2Dh3, and PAdacBΔD. Additionally, we have also shown that this simultaneous increase (on the order of hundreds-fold compared to wild type) necessarily entails a significant increase in the total amount of soluble anhNAM-peptide pool (Fig. 2C). This would contrast with what was proposed in the work of Lee et al. (33), in which, although the AmpC inducers NAG-anhNAM-P5 and anhNAM-P5 experienced a net increase, the whole peptidoglycan-derived soluble fragment pool was shown to be reduced ca. 4-fold upon cefoxitin challenge. These facts suppose another interesting difference between the mechanisms underlying cefoxitin induction and the dynamics of *ampC* hyperexpression in the high-level hyperproducer strains.

Although the two highest hyperproducing mutants, PAΔDDh2Dh3 and PAdacBΔD, display the simultaneous accumulation of anhNAM-P3 and anhNAM-P5 (Fig. 2), the notable quantitative variations of these signals among the two strains did not significantly affect the final outcome of *ampC* expression level (Table 1). This is probably due to the AmpR saturation, supported by the fact that these two strains are not further inducible (57). As stated before, the AmpC-activating power (via AmpR binding) of anhNAM-P5 seems much stronger than that of anhNAM-P3, but in this high-level hyperproduction context, in which the amount of both activators is highly increased (but the number of anhNAM-P3 molecules continues to be much greater than that of anhNAM-P5), it is difficult to predict which species outcompetes the other for AmpR binding, or even if an indistinct binding does occur.

Further analysis of the PAΔDDh2Dh3 mutant helps to explain this simultaneous accumulation of anhNAM-P3 and anhNAM-P5. Since AmpDh2 and AmpDh3 are periplasmic amidases cleaving stem peptides from the peptidoglycan (52–55) and thus initiating the recycling-related role that AmpD exerts within the cytosol, it seems logical that the triple inactivation caused an exaggerated anhNAM-P3 accumulation in comparison with PAΔD (ca. 60-fold higher [Fig. 2A]). A similar conclusion could be deduced with regard to anhNAM-P5: under basal conditions, the amount of this molecule is low (Fig. 1A and B) (33) not only because of the abovementioned d,d-carboxypeptidase activities but also because of stem peptide cleavage by AmpDh2 and AmpDh3. Therefore, the absence of these two proteins should consequently increase the amount of released muropeptides instead of free peptides. In the AmpDh2-AmpDh3 defective background (AmpD still functional), no striking consequences are seen (Fig. 2A and B), suggesting that AmpD is self-sufficient to metabolize this increased amount of cytosolic muropeptides. But when AmpD (the main amidase cleaving the AmpC activators for ulterior anabolism [52, 56, 72]) is absent, the partial or total lack of amidase activity in the periplasm entails a dramatic increase in anhNAM-peptide accumulation and *ampC* expression (47, 56, 57). Despite the high-level β-lactam resistance of PAΔDDh2Dh3, this kind of triple mutant has never been found in nature, likely because it is dramatically impaired in fitness and virulence (73–75). Conversely, PAdacBΔd-type mutants have been readily found in the clinical setting (47). Furthermore, an interesting question arises with this strain: why the activator muropeptide accumulation is much higher (between 10- and 20-fold for anhNAM-P3 and anhNAM-P5) than in the PAΔD and PAΔdB single mutants? The total absence of AmpD in the double mutant (compared to the mere AmpD saturation likely occurring in PAΔdB) could explain the anhNAM-P5 increase. However, the reason why the amount of anhNAM-P3 also increases in PAdacBΔD but is not in appreciable in PAΔdB is more intriguing and could suggest a potential preferential trimming activity of AmpD over anhNAM-P3 versus anhNAM-P5. Finally, here we show that the amount of anhNAM-P4 does not increase in parallel with the *ampC* expression levels in any of the pathways (47, 56, 57) but rather the contrary (Fig. 1B and Fig. 2C), and thus, this muropeptide seems to be expendable for AmpC hyperproduction in P. aeruginosa, in contrast to what has been described for S. maltophilia (46).

It has been previously shown that AmpG inactivation prevents the entrance of NAG-anhNAM-peptides and consequently the cytosolic accumulation of AmpC-activator signals, thereby disabling *ampC* overexpression (47, 76–80). These facts are confirmed by our results, in which *ampG* deletion drastically decreased the intracellular accumulation of anhNAM-P3 and anhNAM-P5 in PAO1 but also in the high-level hyperproducer mutants in comparison with the respective parent strain, which leads to a block of *ampC* expression. Since AmpG inactivation disables the bona fide NAG-anhNAM-peptide cytosolic gate, the still-increased (with regard to PAO1) accumulation of anhNAM-P3 and anhNAM-P5 in PAΔDDh2Dh3ΔAG and PAdacBΔDG (Fig. 3) necessarily corresponds to their increased generation in the periplasm driven by the inactivation of *dacB* and/or *ampD* homologues. One could argue that instead of anhNAM-P3 and anhNAM-P5, in PAΔDDh2Dh3ΔAG and PAdacBΔDG a periplasmic accumulation of NAG-anhNAM-P3 and NAG-anhNAM-P5 should appear, inasmuch as NagZ, responsible for the NAG cleavage, is cytosolic. Nevertheless, as previously proposed (33, 81), the existence of *N*-acetylglucosaminidase activity in the P. aeruginosa periplasm seems highly likely, which would enable the NAG cleavage and explain the large amount of anhNAM-peptides. On the other hand, the previously described increased release of muropeptides to the extracellular medium in AmpG-defective mutants (82–84) could contribute to the reduced amount of soluble anhNAM-peptides of PAΔDDh2Dh3ΔAG and PAdacBΔDG compared to the parent strains (Fig. 3A and B).

In this study, we finally sought to analyze if the peptidoglycan biosynthesis precursor UDP-NAM-P5, which has been reported to act as an AmpC repressor through AmpR binding in different species (17, 19, 32), could have a similar role in P. aeruginosa. Nevertheless, our results suggest that the repressor capacity of UDP-NAM-P5 is fairly minor in P. aeruginosa, since the level of *ampC* expression was generally not inversely proportional to the UDP-NAM-P5 amount in our strains. In fact, in some of our hyperproducer mutants, this precursor was at wild-type (or even higher) levels, a circumstance that has been previously shown for AmpD defective mutants in different species (17). In contrast, our results show a consistent UDP-NAM-P5 reduction in those strains with impaired peptidoglycan recycling, namely, PAΔDDh2Dh3, PAΔDDh2Dh3ΔG, and PAΔAG (74, 75) (Fig. 4). These facts, previously reported in AmpG mutants from other species (17), seem quite logical since recycling disruption blocks a very important pipeline of materials for the anabolism of new UDP-NAM-P5 units. Consistently, PAΔDDh2Dh3ΔG displayed the lowest levels of UDP-NAM-P5 (Fig. 4) given its impairment for both periplasmic turnover (in terms of AmpDh2/AmpDh3-driven stem peptide cleavage that would allow the recycling of the latter) and uptake of NAG-anhNAM-containing fragments via AmpG (25, 34, 36). Therefore, our results do not support a major influence of UDP-NAM-P5 over AmpC regulation in P. aeruginosa. A possible explanation could be that when the activator muropeptides are present, they exert a dominant role, thereby displacing the repressor UDP-NAM-P5 from AmpR (17). In fact, in accordance with this very modest regulatory role that we propose for UDP-NAM-P5, the deletion of AmpR (which eliminates the repression exerted through binding with this precursor) was previously reported to cause only a slight increase of *ampC* expression in P. aeruginosa (below 5-fold compared to wild type) (57).

**FIG 5:**
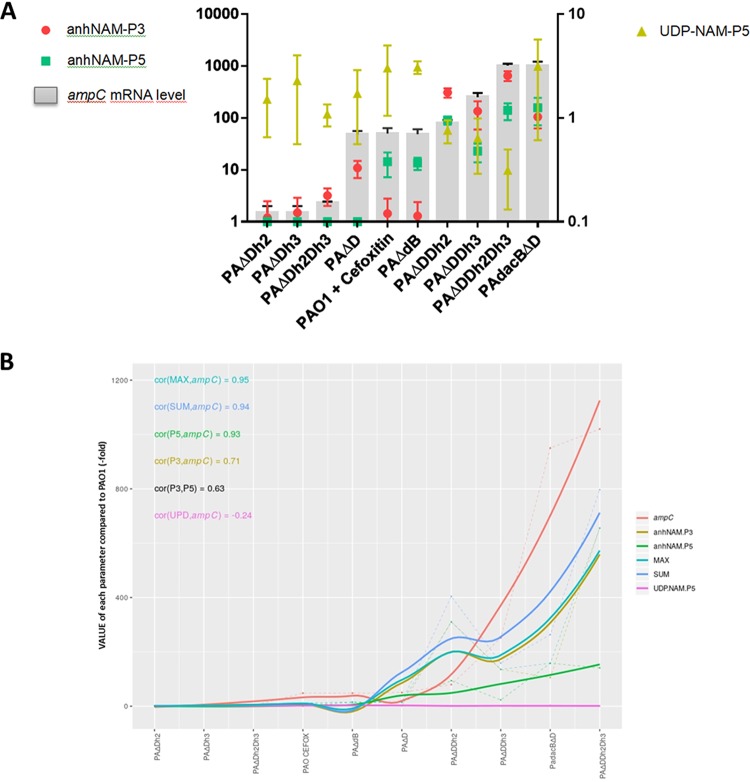
Model for Pseudomonas aeruginosa AmpC derepression, depicting the variations in the pathways (different mutational routes versus cefoxitin induction) and levels of enzyme production, based on the differential accumulation of muropeptides. (A) The gray bars represent the level of *ampC* expression obtained from previous works by our group (52, 53, 74), whereas the red circles, green squares, and yellowish green triangles represent the accumulation of anhNAM-P3, anhNAM-P5, and UDP-NAM-P5, respectively, in each strain (always in terms of fold with regard to PAO1, considered 1). The error bars represent the SD. The scale on the right is valid for only UDP-NAM-P5, whereas that on the left is for the other parameters (both are log scales). (B) Smooth Loess curves of the linear regressions between *ampC* expression and (i) the accumulation of the different independent muropeptides, (ii) the sum of both anhNAM-P3 and anhNAM-P5 (SUM), or (iii) the highest muropeptide of each strain (MAX). The Spearman coefficient for each correlation is shown.

The results we show in this study (summarized in Fig. 5A) allow us to draw a novel model for peptidoglycan-derived signaling enabling *ampC* hyperexpression in P. aeruginosa, which takes into account the different pathways, levels of AmpC production, and the involved activator muropeptides (Fig. 5B). Although with certain quantitative particularities, an excellent correlation (considering Spearman’s coefficients) between the amount of the activator muropeptide anhNAM-P5 and *ampC* expression does exist and is a little weaker for anhNAM-P3. Meanwhile, no correlation with UDP-NAM-P5 is found, indicating a residual role for this molecule as AmpC regulator. Moreover, when applying combinatorial analysis, i.e., choosing the highest value for each strain (anhNAM-P3 or anhNAM-P5), the correlation with *ampC* expression is almost perfect, similar to that obtained by summing the two types of muropeptides, which suggests their additive nature for AmpC activation.

Therefore, our model, which displays certain particularities in comparison with other species’ β-lactamase signaling (16, 17, 42, 43, 45, 46), entails a definitive step to understand the basis for P. aeruginosa AmpC regulation, which could be useful to open new therapeutic conceptions oriented to interfere with the involved peptidoglycan-derived signaling.

## MATERIALS AND METHODS

### Bacterial strains.

A list and description of the strains used in this work are shown in Table 1. P. aeruginosa PAO1 strain-derived single or combined knockout mutants previously constructed according to described procedures (56, 57, 85), based on the Cre-lox system for gene deletion in P. aeruginosa (86), were used. Their data regarding susceptibility to β-lactams, i.e., ceftazidime MICs and *ampC* expression, were obtained from the previous work of our group indicated in Table 1.

### Cefoxitin induction experiments.

For induction experiments, before the realization of each assay, the overnight cultures were diluted 1:50 and were grown in the presence of 50 μg/ml cefoxitin for 3 h (37°C, 180-rpm agitation), as previously described (85).

### Generation of soluble peptidoglycan precursor pools and UPLC-MS analysis.

Sample preparation (at least three independent preparations per strain were done, always referring the data of each determination to the wild type) for collection of cytosolic soluble muropeptides was performed following the protocol described previously by Lee et al. (33) with some modifications. Briefly, bacteria were grown until exponential phase and cooled down on ice for 10 min, and then after adjusting the OD_600_ of all the cultures (to have the same number of bacteria in each sample), normalized volumes of cells were harvested by centrifugation at 4,000 rpm. at 4°C for 20 min. Cell pellets were then gently resuspended, washed with ice-cold 0.9% NaCl solution, and finally resuspended in water and boiled for 15 min. After centrifugation to remove cell debris at 14,000 rpm for 15 min, soluble fractions (containing intracellular soluble muropeptides) were transferred into new tubes and stored at −20°C. Samples were filtered using 0.2-μm-pore-size filters, dried by speed vacuum, resuspended into water, and used for UPLC-MS analyses.

Detection and characterization of soluble muropeptides were performed on a UPLC system interfaced with a Xevo G2/XS quadrupole-time of flight (Q-TOF) mass spectrometer (Waters Corp.). Chromatographic separation was achieved using an Acquity UPLC BEH C_18_ column, 130 Å, 1.7 μm, 2.1 mm by 150 mm (Waters Corp.), heated at 45°C. Formic acid at 0.1% in Milli-Q water (buffer A) and 0.1% formic acid in acetonitrile (buffer B) were used as eluents. The gradient of buffer B was set as follows: 0 to 3 min, 5%; 3 to 6 min, 5 to 6.8%; 6 to 7.5 min, 6.8 to 9%; 7.5 to 9 min, 9 to 14%; 9 to 11 min, 14 to 20%; 11 to 12 min, hold at 20% with a flow rate of 0.175 ml/min; 12 to 12.10 min, 20 to 90%; 12.1 to 13.5 min, hold at 90%; 13.5 to 13.6 min, 90 to 2%, 13.6 to 16 min, hold at 2% with a flow rate of 0.3 ml/min; then 16 to 18 min, hold at 2% with a flow rate of 0.25 ml/min. The Q-TOF MS instrument was operated in positive ionization mode using MSe (mass spectrometry using elevated collision energies). The following parameters were set for electrospray ionization (ESI): capillary voltage at 3.0 kV, source temperature to 120°C, desolvation temperature to 350°C, sample cone voltage to 40 V, cone gas flow of 100 liters/h, and desolvation gas flow of 500 liters/h. Data acquisition and processing were performed using the UNIFI software package (Waters Corp.).

The molecular structure of the soluble muropeptides expected to be found in the cytosolic samples was obtained by using ChemSketch version 14.01 (ACD/Labs, Toronto, Ontario, Canada) and used to build a compound library in UNIFI. This compound library was used for processing the data, detection, and identification of muropeptides. Subsequent confirmation of the structure of automatically detected muropeptide was performed by further analysis of the MS fragmentation pattern and comparison with data previously obtained from the analysis of standard muropeptides. The area of the extracted ion chromatogram of identified muropeptides was considered the quantitative value.

### Data analysis.

GraphPad Prism 5 software was used for graphical representation and statistical analysis. Quantitative variables were compared using two-tailed Student’s *t* test or Mann-Whitney U test as appropriate. A *P* value of <0.05 was considered statistically significant. To find correlations between the accumulation of muropeptides and *ampC* expression, the linear regressions represented by smooth Loess curves were calculated, together with the respective Spearman correlation coefficients, using the R software (version 3.6.0; R Foundation for Statistical Computing, Vienna, Austria).
